# Muscarinic receptor-induced contractions of the detrusor are impaired in TRPC4 deficient mice

**DOI:** 10.1038/s41598-018-27617-5

**Published:** 2018-06-18

**Authors:** Caoimhin S. Griffin, Keith D. Thornbury, Mark A. Hollywood, Gerard P. Sergeant

**Affiliations:** Smooth Muscle Research Centre, Dundalk Institute of Technology, Dublin Road, Dundalk, Co. Louth Ireland

## Abstract

Acetylcholine contracts the bladder by binding to muscarinic M3 receptors on the detrusor, leading to Ca^2+^ influx via voltage-gated Ca^2+^ channels. The cellular mechanisms linking these events are poorly understood, but studies have suggested that activation of TRPC4 channels could be involved. The purpose of this study was to investigate if spontaneous and cholinergic-mediated contractions of the detrusor were impaired in TRPC4 deficient (TRPC4^−/−^) mice. Isometric tension recordings were made from strips of wild-type (WT) and TRPC4^−/−^ detrusor. Spontaneous phasic detrusor contractions were significantly smaller in TRPC4^−/−^ mice compared to wild-type, however no difference in response to exogenous application of 60 mM KCl was observed. Cholinergic responses, induced by electric-field stimulation (EFS), bath application of the cholinergic agonist carbachol, or the acetylcholinesterase inhibitor neostigmine were all significantly smaller in TRPC4^−/−^ detrusor strips than wild-type. Surprisingly, the TRPC4/5 inhibitor ML204 reduced EFS and CCh-evoked contractions in TRPC4^−/−^ detrusor strips. However, TRPC5 expression was up-regulated in these preparations and, in contrast to wild-type, EFS responses were reduced in amplitude by the TRPC5 channel inhibitor clemizole hydrochloride. This study demonstrates that TRPC4 channels are involved in spontaneous and cholinergic-mediated contractions of the murine detrusor. TRPC5 expression is up-regulated in TRPC4^−/−^ detrusor strips, and may partially compensate for loss of TRPC4 channels.

## Introduction

Muscarinic receptor (MR) antagonists are the mainstay treatment for overactive bladder syndrome (OAB)^1^. However, these drugs have a wide range of side effects including blurred vision, cognitive impairment, constipation, and dry mouth^2^, resulting in poor persistence rates. For example, only fifty percent of patients request a repeat prescription following their initial trial of anticholinergic OAB medication and 14–35% of patients continue anticholinergic OAB treatment after one year^2–4^. Therefore, alternative treatments, that inhibit cholinergic responses in the detrusor, but without these side effects, are needed. This requires a better understanding of the mechanisms responsible for mediating acetylcholine (ACh) responses in the bladder which, surprisingly, have still not been elucidated.

Cholinergic-dependent contractions of the bladder are known to be mediated by stimulation of MRs. The M2 and M3 subtypes are most abundant in bladder tissue, however in most species M3Rs are predominantly responsible for muscle contraction^5^. M_3_Rs are coupled to G_q/11_ proteins that activate phospholipase C (PLC) and lead to generation of 1,2-diacylglycerol and inositol-1, 4, 5-triphosphate (IP_3_) yet PLC inhibitors have only modest inhibitory effects on MR-mediated contractions of the detrusor^6–10^. In contrast, it is widely reported, that cholinergic-mediated responses of the detrusor are almost completely dependent on Ca^2+^ influx via voltage-gated calcium channels (VGCC)^6–13^, however the cellular processes that couple stimulation of M3Rs to activation of VGCCs are still unclear.

Recently, Griffin *et al*.^14^, suggested that TRPC4 channels could be involved in this process. They showed that ML204, a drug that inhibits TRPC4 and TRPC5 members of the transient receptor potential canonical (TRPC) channel family, inhibited cholinergic responses in the murine detrusor^14^. PCR experiments revealed that TRPC4, but not TRPC5, was expressed in isolated detrusor myocytes indicating that TRPC4 was likely to be the main TRPC channel involved in this process. Therefore, TRPC4 channels were postulated to be activated following stimulation of M3Rs in detrusor myocytes, leading to depolarisation of membrane potential, and activation of VGCCs. The findings of Griffin *et al*.^14^, opened up the exciting possibility that TRPC4 channels could be targeted for treatment of overactive bladder syndrome (OAB) as a means to inhibit cholinergic responses^15,16^. However, for this to occur, further scrutiny of this idea is required, using alternative experimental approaches that are complimentary to the pharmacological approach employed by Griffin *et al*.^14^.

In the present study we further investigated the idea that TRPC4 channels are involved in cholinergic-mediated contractions of the detrusor by examining if contractile responses to cholinergic agonists and electric field stimulation (EFS) in detrusor strips from TRPC4^−/−^ mice were smaller than those obtained from wild-type litter mates. We also examined if spontaneous contractions were altered in TRPC4^−/−^ detrusor strips and if the expression of other TRPC channel subunits was affected in detrusor preparations lacking functional TRPC4 channels.

## Results

### Comparison of spontaneous and 60 mM KCl-induced contractions of detrusor strips taken from wild type and TRPC4^−/−^ mice

**Figure 1:**
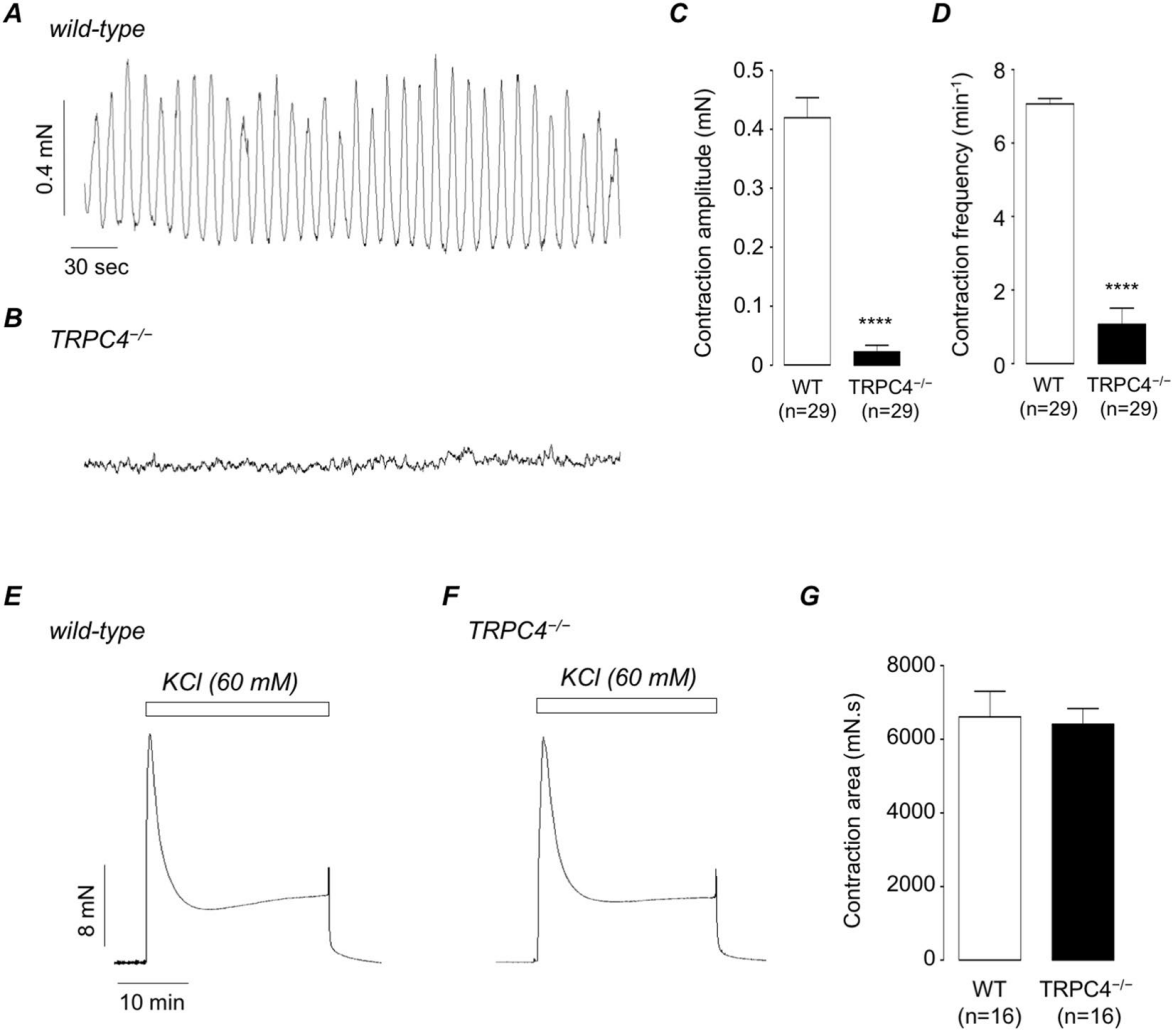
(**A**,**B**) Representative tension recordings of spontaneous activity in detrusor strips taken from wild-type (**A**) and TRPC4^−/−^ mice (**B**). (**C,D**) Summary bar charts showing mean amplitude (**C**) and frequency (**D**) of spontaneous detrusor contractions in wild-type (WT, open bars) and TRPC4^−/−^ mice (filled bars). (**E**,**F**) Representative KCl (60 mM)-evoked contractions in wild-type (**E**) and TRPC4^−/−^ (**F**) detrusor strips. (**G**) Summary bar chart plotting mean KCl-evoked contraction amplitude, (measured as area under curve, mN.s) in wild-type (WT, open bars) and TRPC4^−/−^ detrusor strips. Error bars represent SEM. **** denotes p < 0.0001.

Experiments were originally performed to test if cholinergic-mediated contractions of detrusor strips taken from TRPC4^−/−^ mice were different to wild-type. However, prior to application of any cholinergic agonist or EFS, it became obvious that these strips did not display the typical pattern of spontaneous contractile activity observed in wild-type mice. Figure 1 shows representative recordings from a detrusor strip taken from a wild-type (Fig. 1A) and a TRPC4^−/−^ (Fig. 1B) mouse. Wild-type detrusor strips exhibited spontaneous phasic contractions, but this activity was absent in TRPC4^−/−^ preparations. In 29 wild-type strips (N = 15), spontaneous contractions occurred at a mean frequency of 7.1 ± 0.15 min^−1^ with a mean amplitude of 0.42 ± 0.03 mN (Fig. 1C,D, respectively). In TRPC4^−/−^ detrusor strips this activity was absent, or greatly reduced. Mean frequency was 1.1 ± 0.4 min^−1^ and mean amplitude was 0.02 ± 0.01 mN (P < 0.0001).

To test if these data reflected an impairment in the overall contractile ability of TRPC4^−/−^ detrusor strips, we compared responses to application of 60 mM KCl in both tissues. Figure 1E,F show contractile responses to 60 mM KCl in wild-type and TRPC4^−/−^ detrusor strips, respectively and Fig. 1G is a summary bar chart plotting the mean amplitude of KCl responses in both tissues. The mean amplitude of KCl responses (measured as integrated area under the curve) in wild-type tissues was 6611 ± 693 mN.s, compared to 6412 ± 426 mN.s in TRPC4^−/−^ detrusor strips (n = 16, N = 8 p > 0.05).

### Comparison of cholinergic-mediated contractions of detrusor strips taken from wild-type and TRPC4^−/−^ mice

**Figure 2:**
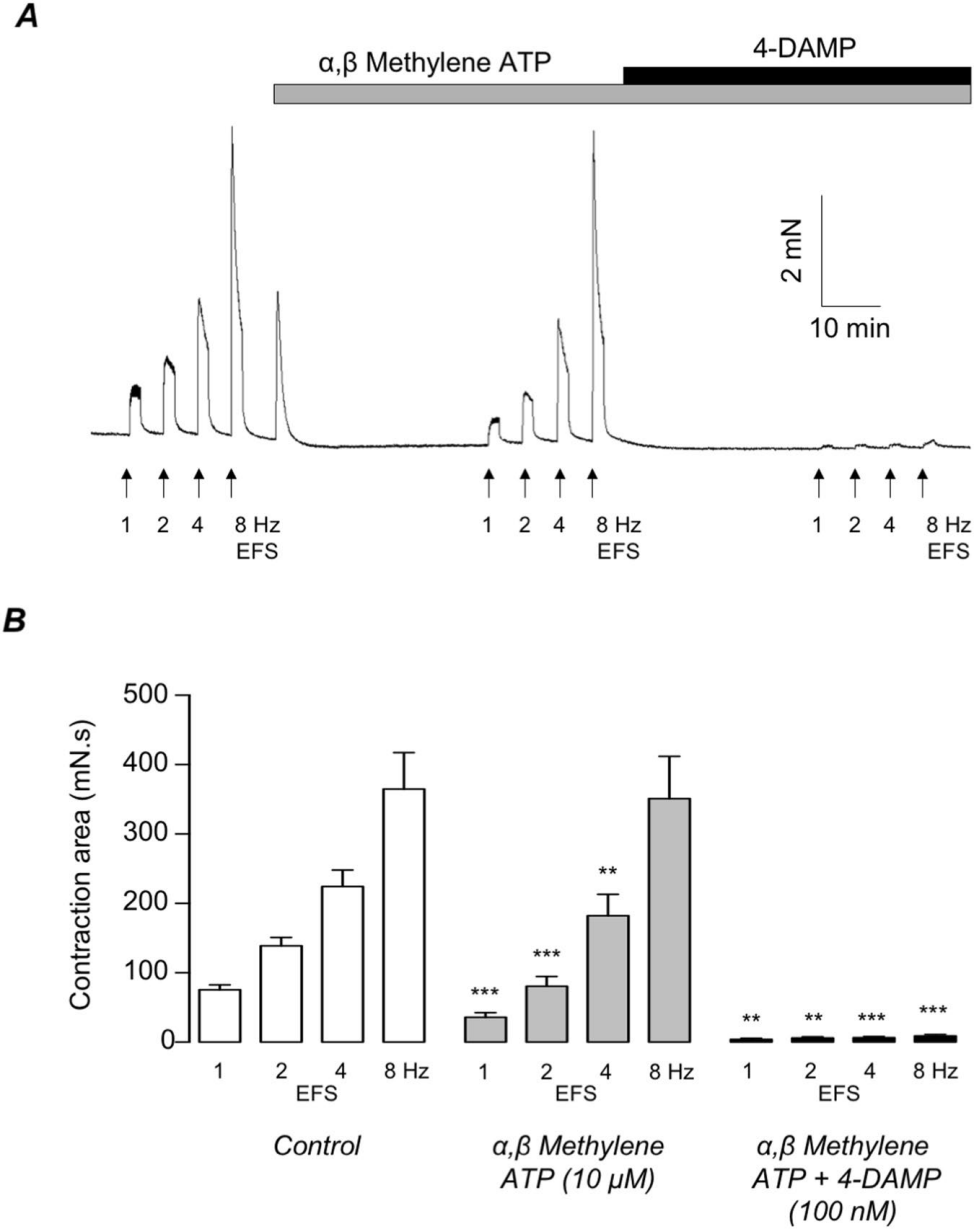
(**A**) Representative traces showing EFS-evoked contractions (1, 2, 4 & 8 Hz, respectively) in TRPC4^−/−^ detrusor strips before, during the presence of α,β-methylene ATP (10 µM) and during the presence of α,β-methylene ATP plus 4-DAMP (100 nM), respectively. (**B**) Summary bar charts showing mean amplitude of EFS-evoked contractions in TRPC4^−/−^ detrusor strips before (open bars), during the presence of α,β-methylene ATP (grey bars) and α,β-methylene ATP plus 4-DAMP (black bars). Error bars represent SEM. ** denotes p < 0.01 and *** denotes p < 0.001, respectively.

Next, we examined if cholinergic responses to EFS (two minutes duration) were impaired in TRPC4^−/−^ detrusor tissues. In order to isolate the cholinergic component of the EFS response these experiments were performed in the presence of 10 µM α,β-methylene ATP to inhibit P2X receptor-dependent responses. Figure 2 shows that responses to 1, 2, 4 & 8 Hz EFS, in the presence of α,β-methylene ATP, were inhibited by the acetylcholine M3R antagonist, 4-DAMP (100 nM), confirming that α,β-methylene ATP-resistant contractions of TRPC4^−/−^ detrusor strips were cholinergic in nature. Figure 2A is a representative trace showing EFS responses, before and during the presence of α,β-methylene ATP and α,β-methylene ATP plus 4-DAMP, respectively. Summary data for 10 experiments (N = 5), showing that 4-DAMP significantly inhibited EFS responses are shown in Fig. 2B. For example, the mean amplitude of contraction, evoked by 2 Hz EFS, was significantly reduced from 139.1 ± 11.8 mN.s, under control conditions, to 80.7 ± 14 mN.s in α,β-methylene ATP (p < 0.001) and 6 ± 1.8 mN.s in α,β-methylene ATP plus 4-DAMP (p < 0.001).

**Figure 3:**
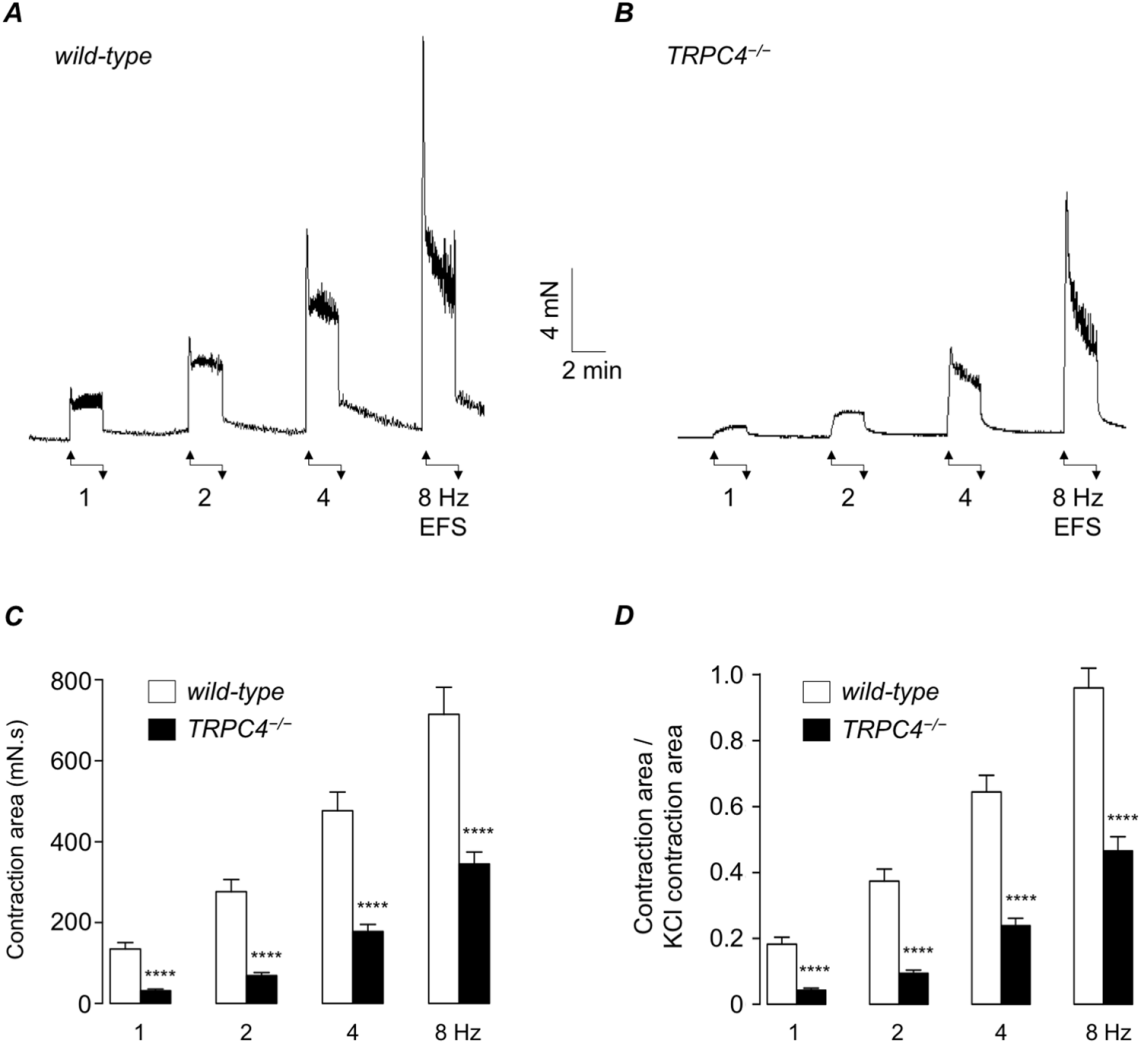
(**A,B**) Representative recordings showing EFS-evoked contractions (1, 2, 4 & 8 Hz, respectively) in wild-type (**A**) and TRPC4^−/−^ detrusor strips (**B**). (**C,D**) Summary bar charts showing mean amplitude of EFS-evoked contractions in wild-type (WT, open bars) and TRPC4^−/−^ preparations (filled bars), raw data (**C**) and normalised to the amplitude of a KCl-induced contraction in the same muscle strip (**D**). Error bars represent SEM. **** denotes p < 0.0001.

The representative recordings in Fig. 3A,B demonstrate that EFS responses (1, 2, 4 & 8 Hz) in TRPC4^−/−^ mice were smaller than those in their wild-type counterparts. Figure 3C plots the mean contraction amplitude at each frequency in both mice. EFS responses at each frequency tested were significantly smaller in TRPC4^−/−^ detrusor tissues compared to wild-type (p < 0.0001, n = 13, N = 7 WT and n = 14, N = 7 TRPC4^−/−^). Figure 3D compares the mean amplitude of EFS responses in both preparations, normalised to the amplitude of a KCl (60 mM) response in each strip. These data also indicate that EFS responses at each frequency were significantly smaller in TRPC4^−/−^ detrusor tissues compared to wild-type (p < 0.0001 n = 13, N = 7 WT and n = 14, N = 7 TRPC4^−/−^).

**Figure 4:**
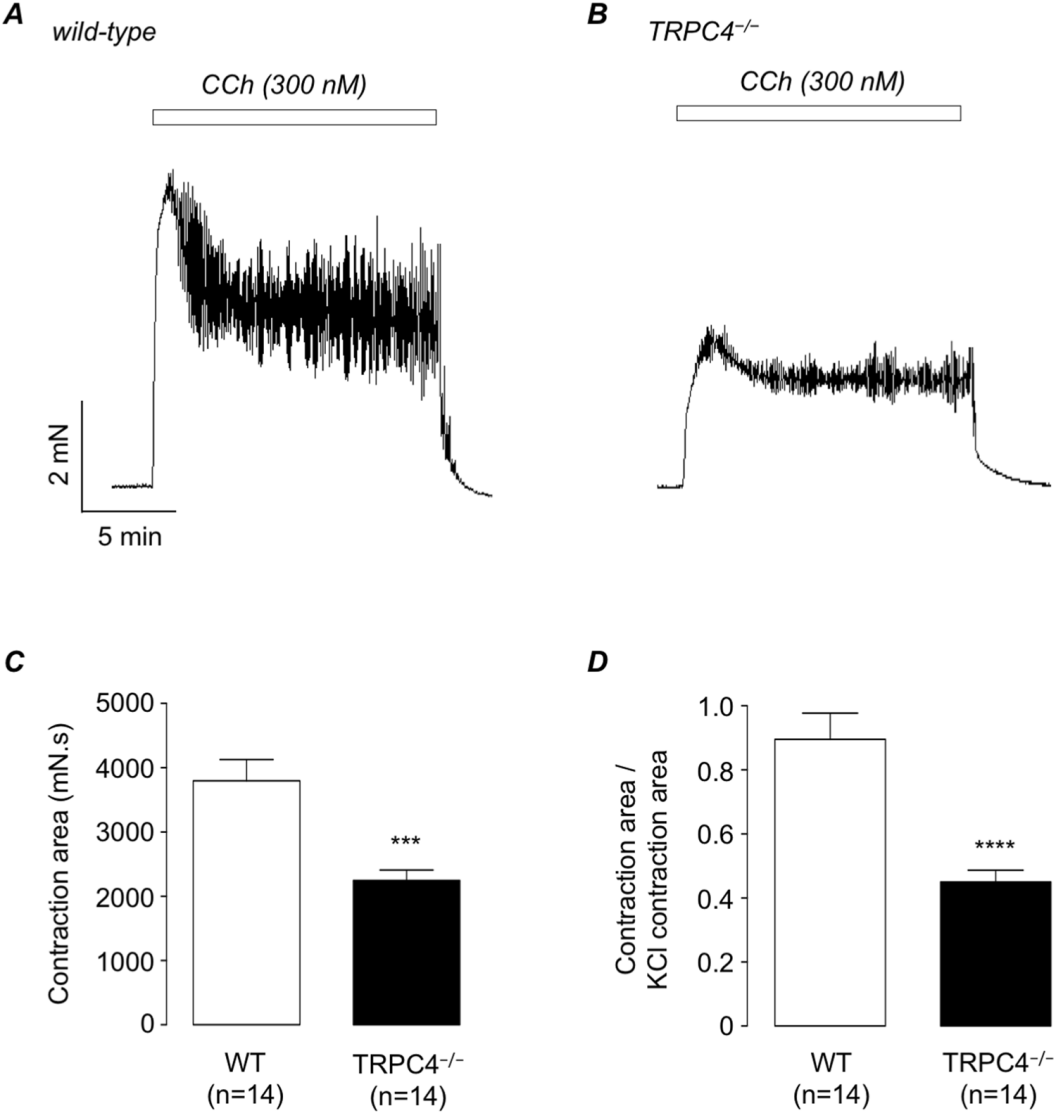
(**A,B**) Representative carbachol (CCh, 300 nM)-induced contraction of wild-type (**A**) and TRPC4^−/−^ detrusor strips (**B**). (**C,D**) Summary bar charts showing mean amplitude of CCh-induced contractions in wild-type (WT, open bars) and TRPC4^−/−^ preparations (filled bars), raw data (**C**) and normalised to the amplitude of a KCl-induced contraction in the same muscle strip (**D**). Error bars represent SEM. *** denotes p < 0.001 and ****p < 0.0001.

Figure 4A,B show representative responses to the cholinergic agonist carbachol (CCh, 300 nM) in wild-type and TRPC4^−/−^ murine detrusor strips. Figure 4C is a plot of the mean amplitude of CCh-induced contractions in both tissue types and Fig. 4D shows these data plotted as a function of the amplitude of a KCl (60 mM) response in each tissue strip. CCh-induced contractions were significantly lower in TRPC4^−/−^ murine detrusor strips than in wild-type (n = 14, N = 7, p < 0.001).

**Figure 5:**
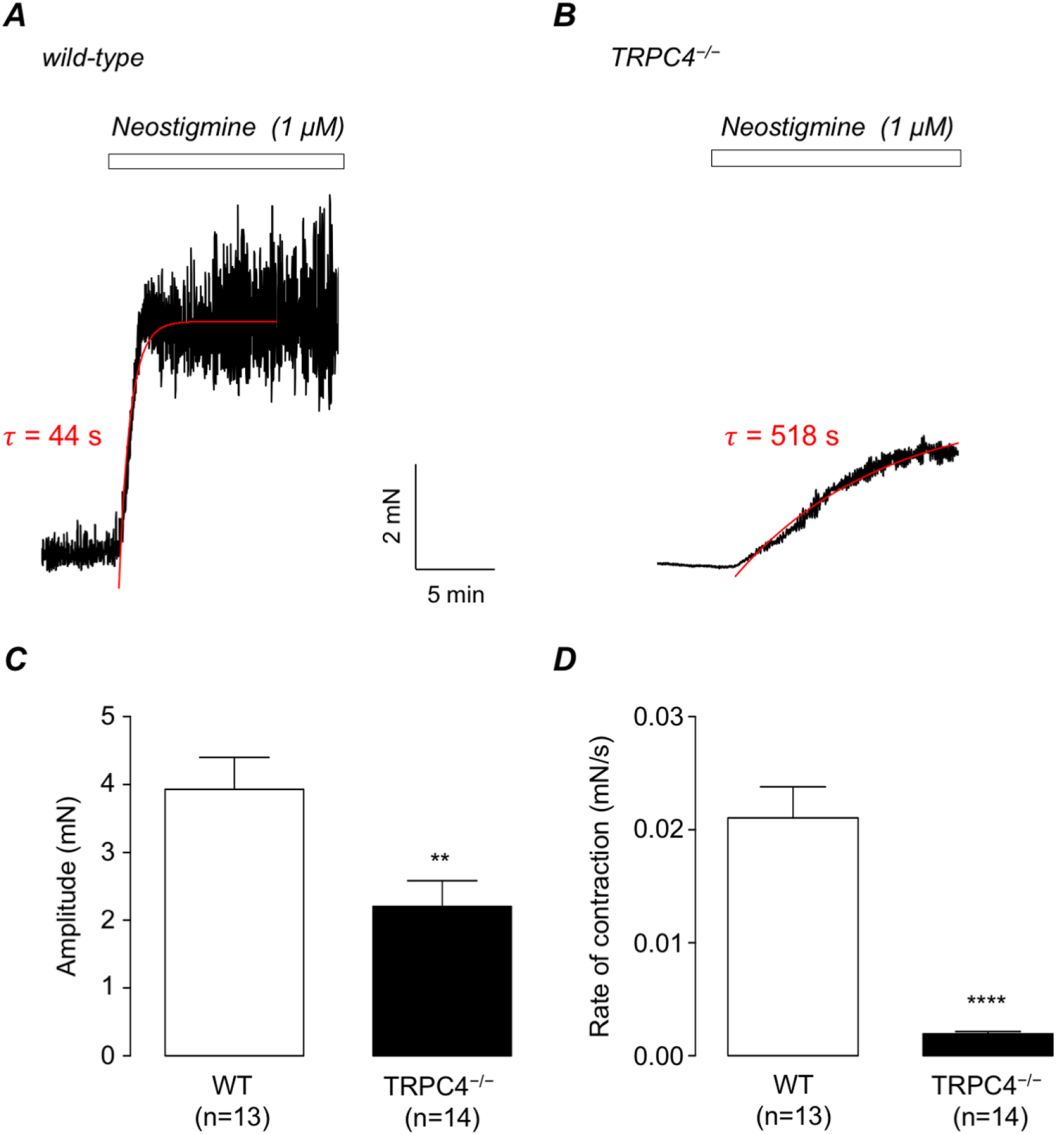
(**A,B**) Representative neostigmine (1 μM)-induced contraction of wild-type (**A**) and TRPC4^−/−^ detrusor strips (**B**). Solid red lines in A&B represent single exponential fits (τ). (**C,D**) Summary bar charts showing mean amplitude of neostigmine-evoked contractions (**C**) and rate of contraction (1/τ) (**D**) in wild-type (WT, open bars) and TRPC4^−/−^ preparations (filled bars). Error bars represent SEM. ** denotes p < 0.01 and ****p < 0.0001, respectively.

Figure 5 shows that responses to the acetylcholinesterase inhibitor, neostigmine (1 μM) were also impaired in TRPC4^−/−^ mice. The amplitude of neostigmine-induced contractions in TRPC4^−/−^ detrusor strips was significantly lower than wild-type controls (Fig. 5C, n = 14, N = 7, p < 0.01). It is also evident from the representative trace in Fig. 5B that there was a notable increase in the time taken to reach the maximal response in TRPC4^−/−^ tissues. Figure 5D shows that the mean rate of contraction (1/τ), induced by neostigmine, using a single-order exponential fit (as indicated by the superimposed solid red lines in Fig. 5A,B) was 0.021 ± 0.0027 mN.s in WT detrusor strips (n = 13, N = 7), compared to 0.0019 ± 0.00019 mN/s in TRPC4^−/−^ detrusor preparations (n = 14, N = 7, p < 0.0001, unpaired *t* test).

### Effect of the TRPC4/5 inhibitor, ML204, on cholinergic-mediated detrusor contractions in wild type and TRPC4^−/−^ mice

**Figure 6:**
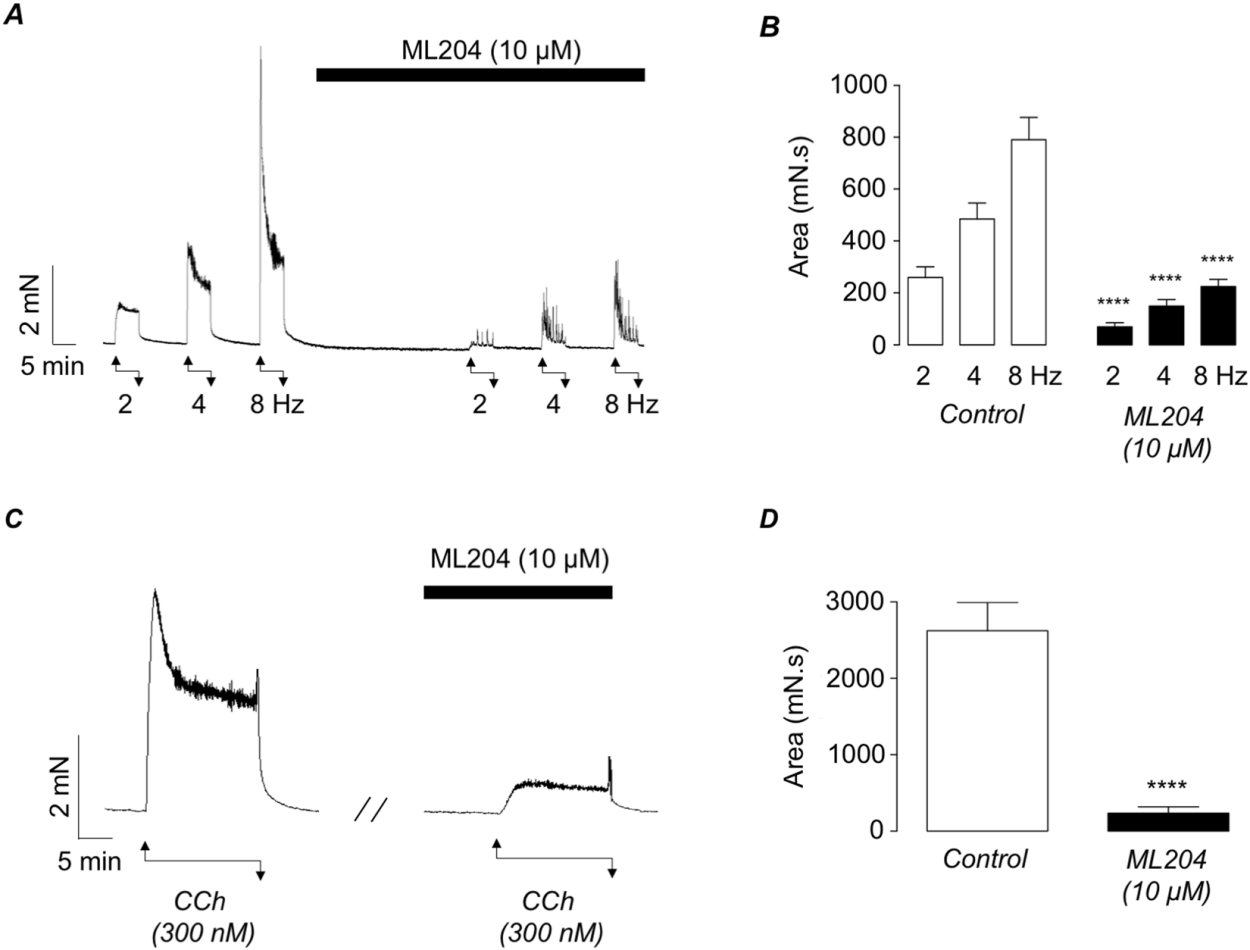
(**A,B**) Representative recording (**A**) and summary bar chart (**B**) showing the effect of ML204 (10 μM) on EFS-evoked contractions (2, 4 & 8 Hz, respectively) in TRPC4^−/−^ detrusor strips. (**C,D**) Representative recording (**C**) and summary bar chart (**D**) showing the effect of ML204 (10 μM) on CCh-induced contractions in TRPC4^−/−^ detrusor strips. Error bars represent SEM. **** denotes p < 0.0001. Note that the duration of the EFS stimulus in these experiments was five minutes, in contrast to two minutes for the experiments shown in Fig. 3.

Griffin *et al*., (2016) demonstrated that the TRPC4/5 inhibitor ML204 inhibited cholinergic-mediated detrusor contractions in wild-type mice^14^. Since TRPC5 was not expressed in detrusor myocytes isolated from wild-type mice this effect was presumed to be mediated by an effect on TRPC4 channels. We reasoned, therefore, that the inhibitory effects of ML204 should be diminished in TRPC4^−/−^ detrusor strips. However, the results shown in Fig. 6A–D demonstrate that ML204 (10 µM) inhibited both EFS and CCh responses in detrusor strips taken from TRPC4^−/−^ mice. CCh-induced contractions were reduced from 2624 ± 369 mN.s to 236 ± 81 mN.s in ML204 (p < 0.0001, n = 12, N = 7). ML204 reduced contractions evoked by 2, 4 and 8 Hz EFS (5 minute duration) from 259 ± 40, 484 ± 61 and 790 ± 86 mN.s to 70 ± 16, 149 ± 25 and 224 ± 28 mN.s respectively (p < 0.0001, n = 13, N = 7).

**Figure 7:**
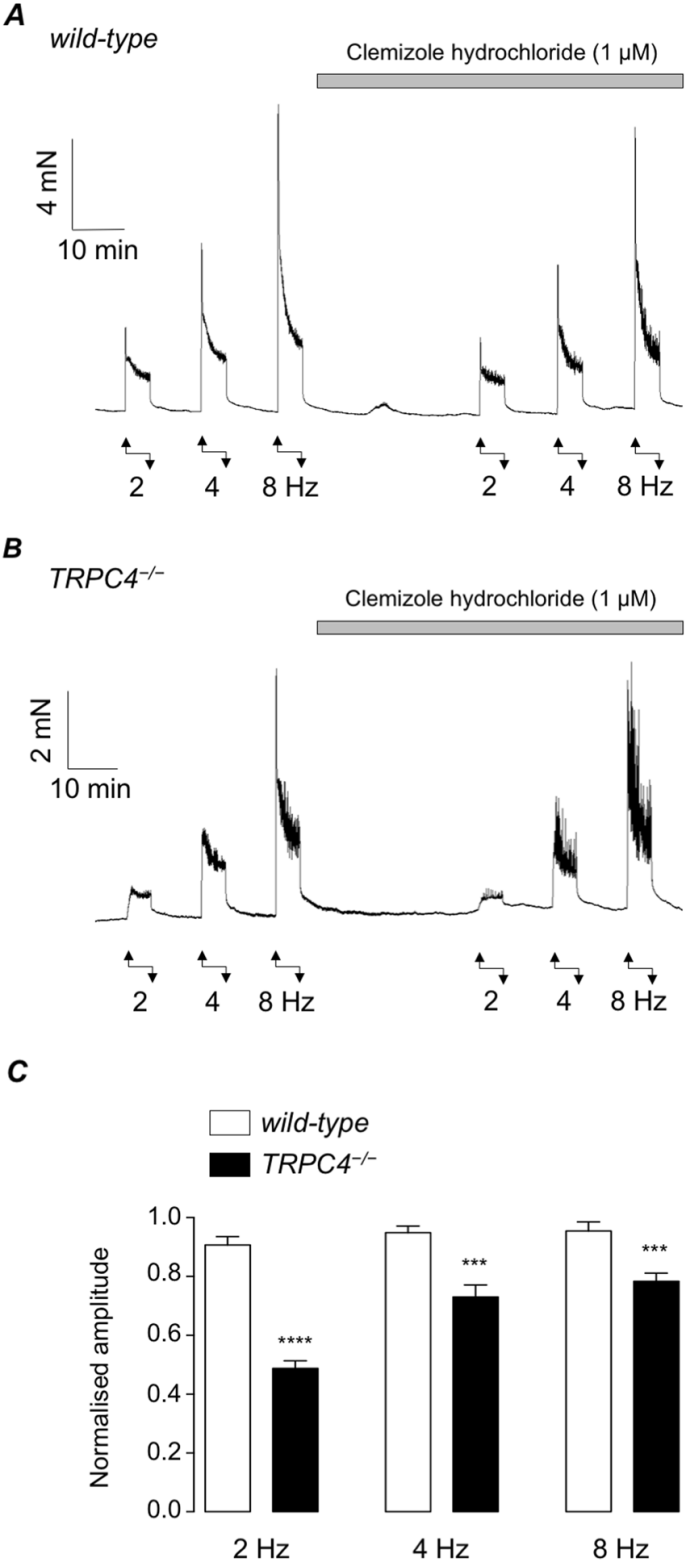
(**A,B**) Representative recordings showing EFS-evoked contractions (1, 2, 4 & 8 Hz, respectively) in wild-type (**A**) and TRPC4^−/−^ detrusor strips (**B**), before and during the presence of the TRPC5 inhibitor, clemizole hydrochloride. (**C**) Summary bar charts showing mean amplitude of EFS-evoked contractions in the presence of clemizole hydrochloride (normalised to the control response at each frequency) in wild-type (open bars) and TRPC4^−/−^ preparations (filled bars). Error bars represent SEM. *** denotes p < 0.001 and **** denotes p < 0.0001.

The effect of clemizole hydrochloride, a TRPC5 channel inhibitor^17^, on EFS responses (2, 4 & 8 Hz) in wild-type and TRPC4^−/−^ detrusor is shown in Fig. 7. Clemizole hydrochloride did not significantly reduce the amplitude of EFS-induced contractions in wild-type detrusor strips (Fig. 7A,C), but induced significant reductions in TRPC4^−/−^ detrusor strips (Fig. 7B,C, n = 12 and N = 6). For example, responses to 2 Hz EFS were significantly reduced by 46% (p < 0.0001) and responses to 4 & 8 Hz EFS were significantly reduced by 23% (p < 0.001) and 18% (p < 0.001), respectively.

**Figure 8:**
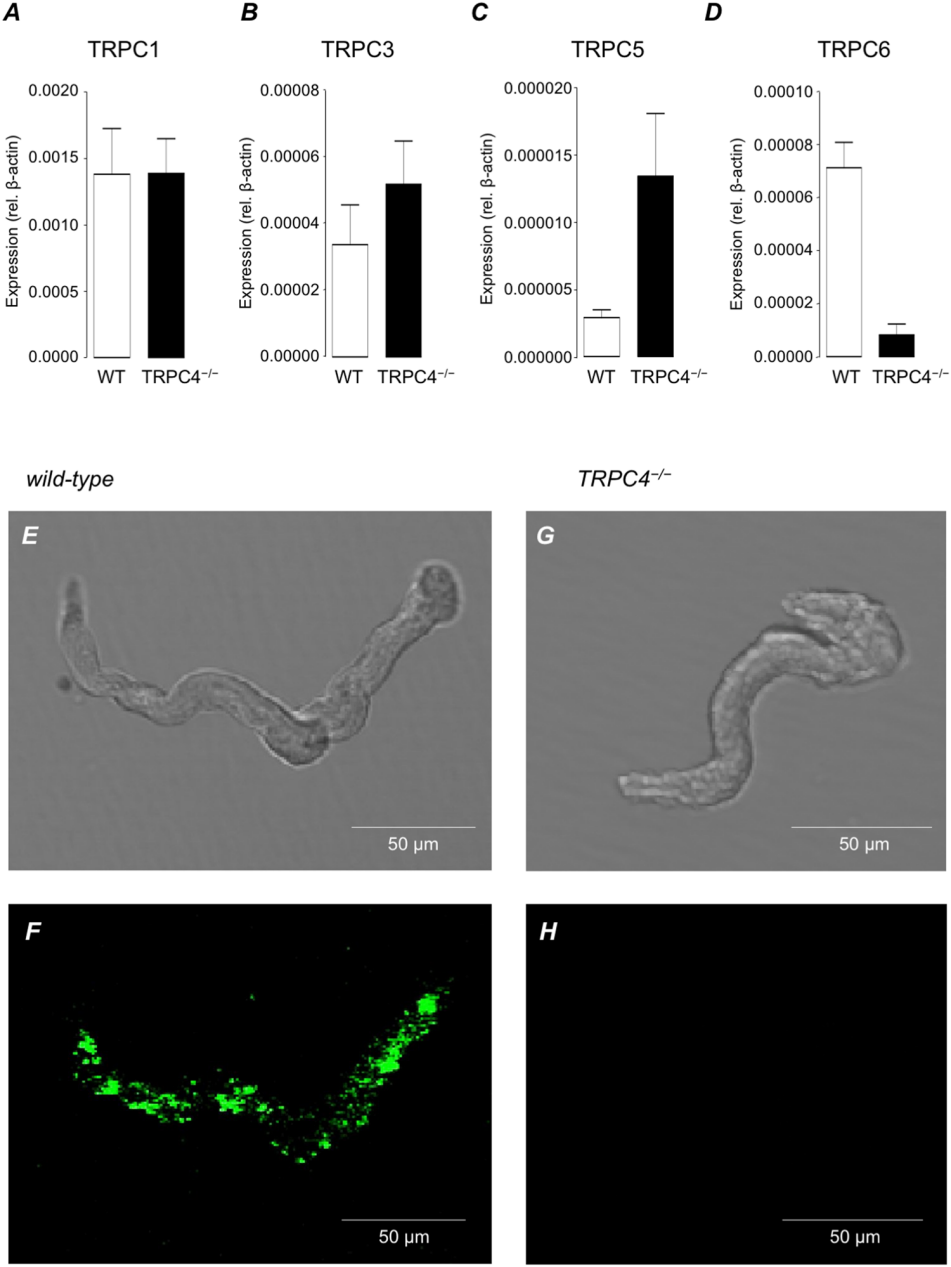
(**A**–**D**) Summary quantitative PCR data showing mean expression levels of TRPC1 (**A**), TRPC3 (**B**), TRPC5 (**C**) and TRPC6 (**D**) (relative to β-actin) in wild-type (WT, open bars) and TRPC4^−/−^ preparations (filled bars). (**E**–**G**) Representative confocal photomicrographs confirming that TRPC4 immunoreactivity was present in freshly isolated smooth muscle cells from wild-type detrusor, but not TRPC4^−/−^ preparations.

Figure 8A–D show the mean expression levels of TRPC1, 3, 5 and 6 in wild-type (N = 8) and TRPC4^−/−^ detrusor (N = 8), relative to the endogenous standard β-actin. Figure 8C shows that the expression levels of TRPC5 in the detrusor is dramatically higher in TRPC4^−/−^ mice compared to wild-type. Interestingly, TRPC6 was notably down-regulated in TRPC4^−/−^ detrusor (Fig. 8D). The representative confocal photomicrographs in Fig. 8E–H confirm that SMC isolated from wild-type murine detrusor (Fig. 8E,F) were immunoreactive for TRPC4, in contrast to those isolated from TRPC4^−/−^ preparations (Fig. 8G,H).

## Discussion

The current study shows that responses to the muscarinic receptor agonist, carbachol, the acetylcholinesterase inhibitor, neostigmine and cholinergic nerve stimulation were significantly smaller in TRPC4^−/−^ mice, compared to wild-type. Furthermore, we also demonstrate that EFS-induced contractions of wild-type detrusor preparations were resistant to application of the TRPC5 channel inhibitor, clemizole hydrochloride. These data follow on from an earlier study by Griffin *et al*., (2016) which showed that cholinergic responses in wild-type mice were inhibited by the TRPC4/5 channel inhibitor ML204^14^. Taken together, these data support the idea that cholinergic-mediated contractions of the detrusor involve activation of TRPC4 channels.

Griffin *et al*.^14^, reported that ML204 reduced the transient and sustained phases of detrusor contractions induced by carbachol and EFS. The remaining oscillatory phase of the contraction was inhibited by the IP_3_R inhibitor 2-APB, indicating that cholinergic contractions of the detrusor had at least two components, mediated by stimulation of TRPC4 channels and IP_3_Rs, respectively. Several studies had indicated that the PLC/IP_3_R pathway accounted for ~25% of the cholinergic response in the detrusor^6–10,18^ but involvement of TRPC4 channels had not been demonstrated previously. Griffin *et al*., (2016) postulated that activation of TRPC4 channels coupled stimulation of M3Rs to Ca^2+^ influx through VGCCs in detrusor myocytes and thus represented a key component of the cholinergic response^14^. However, this conclusion was reliant on the specificity of ML204 and therefore required verification using other experimental approaches. The data contained in the present study provide further support for the idea that TRPC4 channels are key regulators of cholinergic responses in the detrusor. Contractions induced by stimulation of cholinergic nerves and bath application of CCh were reduced by ~50% in TRPC4 deficient mice. Neostigmine responses were reduced in amplitude and were also slower to develop, likely as a function of the time required for the concentration of endogenously released ACh to increase sufficiently to compensate for the reduced expression of TRPC4 channels.

Another interesting finding of the present study is that the spontaneous phasic contractions in the detrusor were also smaller, or absent, in TRPC4^−/−^ mice. This activity, sometimes referred to as the autonomous activity of the bladder^19,20^ seems to be increased in patients with detrusor overactivity^21^. Therefore, this represents another important role for TRPC4 in the bladder and provides an alternative means by which these channels could be targeted to treat OAB. The diminished responses in TRPC4^−/−^ mice appear not to be due to an impairment in the overall contractile activity of these tissues since responses induced by KCl (60 mM) were not affected. This suggests that the activity of VGCCs and the contractile machinery in TRPC4^−/−^ mice was not impaired and therefore was not a contributory factor in the diminished spontaneous and cholinergic-mediated contractions of the TRPC4^−/−^ detrusor.

A surprising, and potentially confounding, finding of the present study is that ML204 still inhibited the cholinergic contractions that were present in TRPC4^−/−^ detrusor preparations. This could indicate that ML204 has non-selective inhibitory effects, and this cannot be ruled out, but since ML204 is recognised as both a TRPC4 and TRPC5 channel blocker we investigated if TRPC5 channel expression was altered in TRPC4^−/−^ detrusor preparations. We found that the expression of TRPC5 increased by ~4 fold in these tissues and thus could, at least partly, account for the inhibitory effects of ML204 in the TRPC4^−/−^ tissues. In support of this idea was the finding that the TRPC5 channel inhibitor, clemizole hydrochloride^17^, reduced the amplitude of EFS responses in TRPC4^−/−^, but not wild-type detrusor strips. The up-regulation of TRPC5 in the TRPC4^−/−^ mice might therefore be a compensatory mechanism in these tissues, however this requires further investigation. This idea is similar to a report by Dietrich *et al*., (2005) which showed that TRPC3 channels were up-regulated in TRPC6 deficient vascular smooth muscle cells, but were not able to functionally replace the TRPC6 channels^22^.

The results of the present study indicate that TRPC4 channels are involved in the post-junctional response to ACh in detrusor myocytes. TRPC4 channels also form part of the signal transduction pathway activated by ACh in ileal SMCs, however, in these cells TRPC4 is thought to act in concert with TRPC6, although the channels are not thought to co-assemble^23^. Interestingly, TRPC6 channels are also expressed in isolated detrusor myocytes^14^ and the present study shows TRPC6 is down-regulated in the TRPC4 deficient detrusor myocytes. This raises the prospect that TRPC6 channels could also be involved in cholinergic responses in the detrusor and that the diminished responses in the TRPC4^−/−^ mice may be related to reduced expression of TRPC6 rather than, or in addition to, reduced TRPC4 expression. Against this idea, however, are findings showing that ML204 is ~19 fold less effective for TRPC6 channels than TRPC4, and that TRPC6 channel inhibition was only detected at concentrations of 20 μM^24^. Griffin *et al*., (2016) previously demonstrated that 1 μM ML204 reduced CCh responses in the murine detrusor by more than 50%^14^, therefore it appears that in wild-type detrusor preparations, at least, TRPC6 channels are unlikely to be involved in the response to activation of M3Rs. However, this requires further investigation. Future studies should also focus on whether TRPC4 expression is altered in OAB or underactive bladder syndrome and if TRPC4 blockers affect voiding contractions *in vivo*.

## Conclusions

Griffin *et al*.^14^, previously demonstrated that; (1) the TRPC4/5 channel blocker ML204 inhibited cholinergic EFS-induced contractions, carbachol-evoked contractions and neostigmine-induced contractions of the murine detrusor and (2) that TRPC4, but not TRPC5, was expressed in isolated murine detrusor myocytes. In the present study we found that; (1) spontaneous contractions of the detrusor were absent in TRPC4^−/−^ mice detrusor (2) contractions induced by carbachol, neostigmine and EFS were smaller in TRPC4^−/−^ detrusor strips, compared to wild-type mice and (3) that the TRPC5 channel blocker clemizole hydrochloride did not affect cholinergic EFS-induced contractions in wild-type detrusor. We believe that, taken together, these data indicate that TRPC4 channels play a crucial role in the response of detrusor myocytes to acetylcholine. However, we cannot rule out the possibility other changes may account for the diminished detrusor responses in TRPC4^−/−^ mice.

## Methods

### Ethical Approval

All procedures were carried out in accordance with EU Directive 2010/63/EU and with approval from Dundalk Institute of Technology Animal Care & Use Committee.

### Tissue preparation

Male and female C57BL/6 (wild-type) mice were obtained from ENVIGO, UK and TRPC4 deficient mice (TRPC4^−/−^) were obtained from the Clapham laboratory, Harvard Medical School, Boston, USA. These mice were generated by use of recombination-mediated genetic engineering techniques, as described by Riccio *et al*.^25^. Mice used in this study were aged between 10–11 weeks old and were killed by cervical dislocation. Bladders were removed, opened longitudinally and pinned to a Sylgard Petri dish with the luminal side facing upwards. The mucosa was removed by sharp dissection, exposing the detrusor. Two strips of detrusor from each bladder were used for tension recording, cell dispersal or molecular biology.

### Tension Recordings

Detrusor strips (~8 mm in length) were mounted in water-jacketed organ baths, maintained at 37 °C and bathed with Krebs’ solution bubbled with 95% O_2_-5% CO_2_. Strips were adjusted to a tension of 2–4mN and allowed to equilibrate for 50 minutes. Contractions were recorded using the multi-channel Myobath system and data acquired using DataTrax2 software (WPI, Europe). Electric field stimulation (EFS) was applied via two platinum electrode wires (5 mm length, 2 mm apart) by a MultiStim system-D330 stimulator (Digitimer Ltd, England), which delivered 0.3-ms pulses of 20 V (nominal) at frequencies 1–8 Hz for 2–5 minutes. These data were analysed using DataTrax2 software. Contraction amplitude in response to EFS and carbachol was determined as the mean integrated tension response, obtained in DataTrax2, by placing cursors at the beginning and end of the stimulus period and measuring the integral of the total area under the trace from the baseline. The rate of contraction was calculated as 1/τ (mN/s). τ was determined by obtaining a single-order exponential fit of the contraction using pClamp software (Molecular devices).

### Molecular Biology

Total RNA was prepared using the Trizol method (Invitrogen). RNA samples were DNase treated (DNase 1, Invitrogen) to remove any contaminating genomic DNA. First-strand cDNA was prepared from RNA using SuperscriptII Reverse Transcriptase (Invitrogen). 200 μg/μl random hexamers were used to reverse transcribe RNA. Real-time quantitative PCR (qPCR) was performed using the SYBR Green PCR Master Mix (Applied Biosystems). TRPC1, 3, 5 & 6 gene specific primer sets were designed using sequences obtained from the Ensembl database. β-actin was used in qPCR as an endogenous reference gene for sample normalization and a β-actin primer set was designed accordingly. The TRPC4 null (^−/−^) mice used in this study contain genetic modification of exon 4 which induces both a frame shift and premature stop codons after the deleted segment. Therefore, the complete TRPC4 transcript is not translated into protein and is not trafficked to the membrane. Therefore, we have opted not to compare TRPC4 transcription levels as they are not representative of protein levels in TRPC4^−/−^ mice. The following PCR primers were used. In each case, the number in parentheses represents the GenBank accession number:

*TRPC1*: Forward - TAGCCCGTCAGTGCAAAATGT

Reverse - TACGGCGGTAACCTGACATC, Amplicon size 252 BP (NM_011643.3).

*TRPC3*: Forward - TGGAAGTTTGCTCGTTCCAA

Reverse - CGAGTTAGACTGTGTGAAGAGG, Amplicon size 219 BP (NM_019510.2).

*TRPC5*: Forward - CCAGAAAGAGTTTGTCGCTCA

Reverse - GATGAAAAGCCCAAGGTTGC, Amplicon size 190 BP (NM_009428.3).

*TRPC6*: Forward - CCTAGCCAGTCAGGAATCTG

Reverse - AATAGTCCTGGCTCTCGTTG, Amplicon size 242BP (NM_013838.2).

### Immunocytochemistry

Strips of detrusor were chopped into pieces (~1 mm^3^) and stored in Ca^2+^-free Hanks’ solution for 30 mins prior to cell dispersal. Tissue pieces were incubated in dispersal medium containing (per 5 ml) of Ca^2+^-free Hanks’ solution:15 mg collagenase (Sigma type 1 A), 1 mg proteinase (Sigma type XXIV), 10 mg bovine serum albumin (Sigma) and 10 mg trypsin inhibitor (Sigma) for 10–15 mins at 37 °C. Tissue was transferred to Ca^2+^-free Hanks’ solution and stirred for 10 mins to release single SMC.

Cells were fixed in 2% paraformaldehyde (PFA, Sigma Aldrich) in phosphate-buffered saline solution (PBS, Gibco), for 30 minutes at 4 °C and washed with PBS (3 × 5 min). This was followed by permeabilisation using Triton X-100 (0.3%, Sigma) and blocking using 3% normal donkey serum (in PBS, 10 mins). Cells were incubated in anti-TRPC4 primary antibody (Alomone Labs, Israel) overnight at 4 °C. The primary antibody was removed by a 3 × 5 minute PBS washing step. Cells were incubated in secondary antibody (Alexa Fluor 488, Invitrogen), 1:1000 dilution for 1 hour at 4 °C. The secondary antibody was removed by a 5 × 5 minute PBS washing step. Secondary antibody control dishes were prepared in similar manner by omitting the primary antibody incubation step. Control dishes were imaged using the same parameters for each experimental. No positive immunoreactivity occurred.

Immunofluorescence of isolated detrusor SMCs was imaged using an Axioskop 2 LSM 510 Meta confocal microscope (Zeiss, Germany). A water-dipping x20 objective lens was lowered into the dish and cells were focused in transmitted light. Using a laser, excitation wavelengths of 488 nm were used to visualise immunoreactivity in the cells. The emission filter used was 505–530 nm (green). Confocal micrographs were composited of z-series scans of ~8–12 optical sections, taken at 0.5 μm depth intervals. Final images were constructed using the z-stack function within ImageJ software.

### Solutions

Solutions used were of the following composition (mM): *Krebs’ Solution*: NaCl (120), KCl (5.9), NaHCO_3_ (25), NaH_2_PO_4_.2H_2_O (1.2), Glucose (5.5), MgCl_2_ (1.2), CaCl_2_ (2.5). pH was maintained at 7.4 by continuous bubbling with 95%O_2–5_% CO_2_. *Ca*^*2+*^*-free Hanks’ Solution (Cell Isolation)*: NaCl (125.0), KCl (5.4), Glucose (10.0), Sucrose (2.9), NaHCO_3_ (15.5), KH_2_PO_4_ (0.4), NaH_2_PO4 (0.3), HEPES (10.0) pH to 7.4 using NaOH. *Ca*^*2+*^*-free Hanks’ Solution (Superfusion)*: NaCl (125.0), KCl (5.4), Glucose (10.0), Sucrose (2.9), NaHCO_3_ (4.2), KH_2_PO_4_ (0.4), NaH_2_PO_4_ (0.3), MgCl_2_.6H_2_O (2.3), EGTA (5.0), MgSO_4_ (0.4), HEPES (10.0). pH to 7.4 using NaOH. *Hanks Solution*: NaCl (125.0), KCl (5.4), Glucose (10.0), Sucrose (2.9), NaHCO_3_ (4.2), KH_2_PO_4_ (0.4), NaH_2_PO_4_ (0.3), MgCl_2_.6H_2_O (0.5), CaCl_2_.2H_2_O (1.8), MgSO_4_ (0.4), HEPES (10.0). pH to 7.4 using NaOH.

### Drugs

ML204 (4-Methyl-2-(1-piperidinyl)-quinoline), α,β-Methylene ATP and clemizole hydrochloride (Tocris), neostigmine & carbachol (Sigma).

### Statistics

In each data set ‘n’ reflects the number of cells or tissue strips and ‘N’ refers to the number of animals from which these were obtained. A maximum of 2 detrusor strips were obtained from each animal. Statistical analysis of responses in wild-type and TRPC4^−/−^ mice was performed using Student’s unpaired *t*-test. The datasets generated during and/or analysed during the current study are available from the corresponding author on reasonable request.

## References

[1] Izett M, Zacche M, Thiagamoorthy G, Robinson D, Cardozo L (2017). Current evidence and emerging drug therapies for overactive bladder. Minerva Ginecol..

[2] Wagg A, Compion G, Fahey A, Siddiqui E (2012). Persistence with prescribed antimuscarinic therapy for overactive bladder: a UK experience. BJU Int..

[3] Sexton CC (2011). Persistence and adherence in the treatment of overactive bladder syndrome with anticholinergic therapy: a systematic review of the literature. Int J Clin Pract..

[4] Thiagamoorthy G, Cardozo L, Robinson D (2016). Current and future pharmacotherapy for treating overactive bladder. Expert Opin Pharmacother..

[5] Andersson KE, Arner A (2004). Urinary bladder contraction and relaxation: physiology and pathophysiology. Physiol Rev..

[6] Schneider T, Fetscher C, Krege S, Michel MC (2004). Signal transduction underlying carbachol-induced contraction of human urinary bladder. J Pharmacol Exp Ther..

[7] Wegener JW (2004). An essential role of Cav1.2 L-type calcium channel for urinary bladder function. FASEB J..

[8] Frei E, Hofmann F, Wegener JW (2009). Phospholipase C mediated Ca^2+^ signals in murine urinary bladder smooth muscle. Eur J Pharmacol..

[9] Frazier EP, Braverman AS, Peters SL, Michel MC, Ruggieri MR (2007). Does phospholipase C mediate muscarinic receptor-induced rat urinary bladder contraction?. J Pharmacol Exp Ther..

[10] Nausch B, Heppner TJ, Nelson MT (2010). Nerve-released acetylcholine contracts urinary bladder smooth muscle by inducing action potentials independently of IP3-mediated calcium release. Am J Physiol Regul Integr Comp Physiol..

[11] Fovaeus M, Andersson KE, Batra S, Morgan E, Sjögren C (1987). Effects of calcium, calcium channel blockers and Bay K 8644 on contractions induced by muscarinic receptor stimulation of isolated bladder muscle from rabbit and man. J Urol..

[12] Ekman M, Andersson KE, Arner A (2009). Signal transduction pathways of muscarinic receptor mediated activation in the newborn and adult mouse urinary bladder. BJU Int..

[13] Wuest M (2007). Contribution of Ca^2+^ influx to carbachol-induced detrusor contraction is different in human urinary bladder compared to pig and mouse. Eur J Pharmacol..

[14] Griffin CS (2016). Muscarinic Receptor Induced Contractions of the Detrusor are Mediated by Activation of TRPC4 Channels. J Urol..

[15] Chess-Williams R (2016). Editorial comment. J Urol..

[16] Michel M (2016). Editorial Comment. J Urol..

[17] Richter JM, Schaefer M, Hill K (2014). Clemizole hydrochloride is a novel and potent inhibitor of transient receptor potential channel TRPC5. Mol Pharmacol..

[18] Herrera GM, Etherton B, Nausch B, Nelson MT (2005). Negative feedback regulation of nerve-mediated contractions by KCa channels in mouse urinary bladder smooth muscle. Am J Physiol Regul Integr Comp Physiol..

[19] Drake MJ, Harvey IJ, Gillespie JI (2003). Autonomous activity in the isolated guinea pig bladder. Exp Physiol..

[20] Gillespie JI (2004). The autonomous bladder: a view of the origin of bladder overactivity and sensory urge. BJU Int..

[21] Drake MJ, Harvey IJ, Gillespie JI, Van Duyl WA (2005). Localized contractions in the normal human bladder and in urinary urgency. BJU Int..

[22] Dietrich A (2005). Increased vascular smooth muscle contractility in TRPC6^−/−^ mice. Mol Cell Biol..

[23] Tsvilovskyy VV (2009). Deletion of TRPC4 and TRPC6 in mice impairs smooth muscle contraction and intestinal motility *in vivo*. Gastroenterology..

[24] Miller M (2011). Identification of ML204, a novel potent antagonist that selectively modulates native TRPC4/C5 ion channels. J Biol Chem..

[25] Riccio A (2014). Decreased Anxiety-Like Behavior and Gαq/11-Dependent Responses in the Amygdala of Mice Lacking TRPC4 Channels. J Neurosci..

